# Effect of Oils Extracted from Plant Seeds on the Growth and Lipolytic Activity of *Yarrowia lipolytica* Yeast

**DOI:** 10.1007/s11746-017-2975-1

**Published:** 2017-03-25

**Authors:** Jolanta Krzyczkowska, Mariola Kozłowska

**Affiliations:** Department of Chemistry, Faculty of Food Sciences, Warsaw University of Life Sciences (WULS-SGGW), Nowoursynowska 159c St., 02-776 Warsaw, Poland

**Keywords:** *Yarrowia lipolytica*, Lipolytic activity, Plant seed oil, Soxhlet method, Folch method

## Abstract

This study was aimed at evaluating the capability of *Yarrowia lipolytica* W29 for the synthesis of lipolytic enzymes in a medium containing plant oils from non-conventional sources with some components displaying bioactivity. Oils from almond, hazelnut, and coriander seeds were obtained by using *n*-hexane (Soxhlet method) and a chloroform/methanol mixture of solvents (Folch method), and their effect on the growth and lipolytic activity of *Y. lipolytica* was compared. A comparison of these two extraction methods showed that the extraction with *n*-hexane was less effective regarding the oil extraction yields than the extraction conducted according to Folch’s procedure. The lipolytic activity of the studied yeast was higher in the culture media containing oils extracted with the Soxhlet method than the Folch method but it was lower compared to olive oil medium. Among all oils tested, almond oil extracted with *n*-hexane was the best inducer of extracellular lipases synthesized by *Y. lipolytica*. Its lipolytic activity achieved the maximum value of 2.33 U/mL after 48 h of culture. After 24 h of culture, it was close to the value obtained for the medium containing olive oil. Almond oil was a source of oleic and linoleic acids, which may determine differences in the lipolytic activity. The linoleic acid content in almond oil was higher than that found in other oils. When n-hexane was used for extraction, the resultant oils were characterized by lower contents of polyphenols and poorer antioxidative activity.

## Introduction

*Yarrowia lipolytica* has been one of the most extensively studied yeast species in recent years. It is a strictly aerobic species that produces many important metabolites and has a high secretory capacity. This makes it important from the biotechnological perspective. Ample studies have been conducted, for years, on the use of *Y. lipolytica* for the synthesis of organic acids, sweeteners, carotenoids, microbial oil, and flavoring compounds [1–3]. One of the most important products secreted by *Y. lipolytica* is lipase, which is a very attractive enzyme for many commercial applications such as the production of detergents, pharmaceuticals, flavoring compounds, dietary lipids, special-purpose products, and bioemulsifiers. Lipases (E.C.3.1.1.3) which are also called glycerol ester hydrolases, are responsible for the hydrolysis of triglycerides into free fatty acids, mono- and diacylglycerols, and glycerol. Using different substrates, they catalyze reactions of hydrolysis, esterification and transesterification including acidolysis, interesterification, and alcoholysis. A growing interest has recently been observed in lipases, mainly due to their wide biotechnological applicability, especially in the food technology, fine chemical synthesis, and the biodiesel industry [4].

The lipolytic activity of *Y. lipolytica* was described for the first time by Peters and Nelson, but the genes encoding proteins with lipase activity were discovered in the last two decades [5–7]. There are two fractions of *Y. lipolytica* lipases: extracellular and intracellular ones (enzymes located in cytosol and connected with structures of a cell wall) that are encoded by the family of *LIP* genes [8]. The course of lipase biosynthesis by *Y. lipolytica* and the catalytic activity of lipases are affected by many factors. One of the key ones is the composition of a culture medium, mainly carbon and nitrogen sources, and the presence of inducing substances. An important role is also ascribed to conditions for microbial growth, such as temperature, pH of the medium, and concentration of dissolved oxygen. By optimizing parameters of lipase synthesis, it is possible to influence not only the amount of synthesized enzymatic protein, but also its activity and, consequently, the price of the resultant preparation. Hydrophobic substrates are considered to be the most efficient carbon sources, which stimulate the production of lipolytic enzymes by microorganisms. Among them, great attention has been paid to vegetable oils (olive oil, sunflower, corn, palm, rapeseed, soybean oil) and pure fatty acids (palmitic acid, oleic acid) [9]. Olive oil is an excellent carbon source to enhance lipase synthesis also by *Y. lipolytica.* It is associated by means of its high content of oleic acid, which is also considered to be an inducer of the *LIP2* promoter [9, 42].

In the present study, we became interested in the feasibility of using lesser known oils from non-traditional sources such as oils obtained from almond, hazelnut, and coriander seeds in the synthesis of lipases by *Y. lipolytica*. They were chosen for the study because of their reported high content of unsaturated fatty acids, including oleic and linoleic acids, which are considered to be inducers of lipolytic activity [10]. Furthermore, at our laboratory, we are carrying out research on the isolation of lipoxygenase (an enzyme involved in the synthesis of green note aroma compounds) from these seeds [11]. Considering the above, it is quite significant and valuable from the economical point of view to manage the residues of plant material after enzyme extraction in the future. The growth of *Y. lipolytica* and its lipolytic activity were assessed in culture media supplemented with plant oils obtained from seeds by extraction with *n*-hexane (Soxhlet method) and a chloroform/methanol mixture of solvents (Folch method). Hexane is a solvent with a high volatility, a low sensitivity to heat, and is relatively easy to remove from the solids and oil at low energy consumption. It is also an ideal solvent for the extraction of non-polar lipids. In turn, Folch’s method is a simple method used in the isolation and purification of total lipids from tissues (both non-polar and polar). The oils extracted with the Folch’s method may also be characterized by a higher oxidative stability compared to oils extracted with the Soxhlet’s method. It would be due to the protective effect of natural antioxidants (phenolic compounds) and phospholipids which are more extractable with a polar solvent. Therefore, the objective of this study was to obtain the oils from almond, hazelnut and coriander seeds by extraction with the Soxhlet and Folch methods, and evaluate their use as a carbon source to evaluate and compare the effect on the growth and lipolytic activity of *Y. lipolytica*. The lipolytic activity of this yeast was also investigated in regard to the culture medium containing olive oil that is often used as a hydrophobic carbon source in microbiological lipase production. The analyzed oils were, additionally, determined for fatty acid composition, total phenolic content, and the ability to scavenge DPPH radicals.

## Materials and Methods

### Materials and Chemicals

Coriander (*Coriandrum sativum* L.) seeds were purchased from a local food store in Warsaw, Poland, whereas almonds (*Prunus dulcis*) and hazelnuts (*Corylis avellana* L.) were obtained from the Hebar Company, Poland. The yeast strain of *Y. lipolytica* W29 (ATCC20460) was obtained from the culture collection of the Food and Microbiology Processing and Engineering Laboratory (GPMA) at the University of Burgundy/AgroSup, France. The strain was stored in 10% glycerol in freezing conditions (−80 °C). All medium ingredients (agar–agar, yeast extract, peptone, and glucose) were obtained from BTL Łódź, Poland. Sodium chloride, methyl and ethyl alcohol (99.8%), chloroform, ethyl acetate, *n*-hexane, toluene, and anhydrous magnesium sulfate were purchased from POCH, Poland, a division of Avantor Performance Products (Center Valley, PA, USA). Trolox (6-hydroxy-2,5,7,8-tetramethylchroman-2-carboxylic acid), DPPH (2,2-diphenyl-1-picrylhydrazyl), Folin-Ciocalteu’s phenol reagent, and gallic acid were obtained from Sigma-Aldrich Chemicals, Poznan, Poland. *p*-Nitrophenyl laurate was synthesized at our laboratory from lauroyl chloride (Sigma-Aldrich) and *p*-nitrophenol (POCH), according to a method described by Vogel *et al*. [12]. All reagents used were of analytical grade.

### Seed Oil Extraction

#### *n*-Hexane Extraction

Plant seeds were crushed and ground into a fine powder. The oil was extracted from each seed sample (30.0 g) with *n*-hexane (250 mL) using a Soxhlet extractor at 70 °C for 6 h. After extraction, the hexane-oil mixture was passed through a layer of anhydrous magnesium sulfate placed over filter paper in a funnel. The solvent was evaporated under vacuum using a rotary evaporator at 40 °C. The resultant oil was weighed and flushed with nitrogen, and stored at −20 °C until further analysis. The oil extraction yields were as follows: hazelnut oil −56.65% ± 0.35, almond oil −43.75% ± 0.77, and coriander oil −16.60% ± 0.20.

#### Chloroform/Methanol Extraction

Forty grams of ground plant seeds were mixed with a solution of chloroform and methanol (2:1, v/v, 300 mL) according to Folch *et al*. [13]. Then, the mixture was filtered through a paper filter into a separatory funnel, and potassium chloride (70 mL, 0.9%) was added. After gentle shaking, the mixture was allowed to separate into two layers. The lower layer was collected and solvents were evaporated under vacuum at 40 °C. The resultant oil was flushed with nitrogen and stored at −20 °C until further analysis. The oil extraction yields were as follows: hazelnut oil −57.50% ± 0.50, almond oil −50.24% ± 0.86, and coriander oil −20.32% ± 0.30.

#### Culture Conditions

A preculture of *Y. lipolytica* W29 was prepared in two steps. *Y. lipolytica* strain was cultured for 48 h on Petri dishes with the YPDA medium (Yeast Extract-Peptone-Glucose-Agar—yeast extract 10 g/L, peptone 20 g/L, glucose 20 g/L, agar 20 g/L) at 28 °C and used to inoculate 100-mL baffled Erlenmeyer flasks containing 20 mL of the YPD medium (yeast extract 10 g/L, peptone 20 g/L, glucose 20 g/L). The flasks were shaken in a KS 4000 ic control shaker (IKA^®^-Werke, Staufen, Germany) at 140 rpm at 28 °C for 24 h. Afterwards, 1 mL of this medium was added to a 500-mL Erlenmeyer flask containing 200 mL of the cultivation medium YP (yeast extract 10 g/L and peptone 20 g/L) with 1% content of different carbon sources (olive oil, oil extracted from almonds, hazelnuts or coriander seeds), and was grown at 28 °C and 140 rpm for 96 h. Yeast cultures were done in three replicates.

#### Measurement of Optical Density

The optical density (OD) of the yeast culture was determined using a Helios Gamma UV–Vis spectrophotometer (Thermo Scientific, Waltham, MA, USA) at a wavelength of 600 nm. To this end, 1 mL of the culture medium was centrifuged using an Eppendorf microcentrifuge, model 5418 (ROTH). The supernatant was decanted, and the cells were suspended in 1 mL of distilled water. The optical density was measured after appropriate dilution of the prepared sample (OD between 0.7 and 1.3).

#### Evaluation of Yeast Growth in the Bioscreen C Analyzer

Growth curves of the studied yeast strain were plotted by an automatic growth analysis system BIOSCREEN C by measuring the optical density at 600 nm in a 96-hour shaking culture at 28 °C, in a well microplate (Oy Growth Curves, Helsinki, Finland), recording the absorbance value every 1 h. For measurement, 0.3 mL of a medium inoculated with 24-h inoculation culture was added to each cell in the microplate. The optical density of the medium without inoculated yeast culture was measured as well (blank test).

#### Determination of Lipase Activity

In order to determine the lipolytic activity, the spectrophotometric method was used to measure the progress of hydrolysis of *p*-nitrophenyl laurate at a wavelength of 410 nm [14]. The amount of the enzyme capable of releasing 1 µmol of *p*-nitrophenol per minute under the assay conditions at 37 °C was accepted as the unit of the lipase enzymatic activity (*U*). Briefly, 0.3 mM of the substrate (*p*-nitrophenyl laurate) was dissolved in 2 mL of heptane and a biocatalyst was added (i.e., 15 mL of the supernatant). The reaction was carried out for 3 and 15 min on a Heidolph MR Hei magnetic stirrer (Heidolph Instruments Labortechnik, Schwabach, Germany)—using a standard function of heating at 37 °C. Afterwards, 100 μL of the reaction mixture was transferred to 3 mL of a 0.1 M sodium hydroxide solution, and the measurement was made in a spectrophotometer at a wavelength of 410 nm. In addition, a blank test was performed for the reaction mixture without substrate addition.

#### Determination of Fatty Acids in Plant Oils

Fatty acid composition was analyzed using gas chromatography after derivatization to fatty acid methyl esters with a methanolic solution of potassium hydroxide according to the standard method [15]. A Shimadzu GC17A gas chromatograph (Shimadzu Corporation, Kyoto, Japan) equipped with a flame ionization detector and a BPX capillary column (30 m × 0.22 mm × 0.25 µm) was applied. The analysis was performed using nitrogen as a carrier gas and applying the following temperature program: 60 °C for 1 min, after which the temperature was increased to 170 °C at a rate of 10 °C/min and from 170 to 230 °C at a rate of 3 °C/min. The temperature of 230 °C was kept for another 15 min. Injector and detector temperatures were 225 and 250 °C, respectively. Column flow rate was set at 1 mL/min with a split ratio 50:1. Identification of fatty acids was based on peak retention time by comparison with the retention time of standard samples and quantified as a percentage of the total fatty acids.

#### Determination of Total Phenolic Content in Methanolic Extract

One gram of oil sample was dissolved in 5 mL of *n*-hexane, and 5 mL of methanol was added. The mixture was vigorously vortexed and centrifuged at 7500 rpm (Biofuge Stratos, Thermo Fisher Scientific, Waltham, MA, USA) for 5 min (6,170*g*, max. radius 9.8 cm). The methanolic layer was separated from the lipid phase, and the extraction with a new portion of methanol was repeated. The total phenolic content in the methanolic extracts was determined using a Folin-Ciocalteu’s reagent [16]. Briefly, 0.5 mL of the methanolic extract was diluted in water and then the Folin-Ciocalteu’s reagent (0.5 mL) was added. After 3 min, 1 mL of a sodium carbonate solution (20%) was added, and the absorbance was measured at 760 nm after 60 min with the samples standing in the dark. The total phenolics content in each sample was determined using a standard curve plotted for gallic acid. The results were expressed as µg gallic acid per gram of oil.

#### DPPH Radical Scavenging Activity

The antioxidant activity of seed oil samples and their methanolic extracts was determined using DPPH radicals [17]. The methanolic extracts (0.5 mL) were diluted with methanol, and 0.25 mL of a freshly prepared 1 mM methanolic solution of DPPH was added. The samples were vortexed and after 10 min the absorbance was measured at 515 nm. The results were expressed in Trolox equivalent antioxidant capacity using a Trolox calibration curve. In order to determine antiradical activity of seed oil samples, 50 mg of each oil were dissolved in 3 mL of ethyl acetate. Then, 1 mL of an oil solution was diluted with ethyl acetate, and 0.25 mL of a freshly prepared DPPH solution (1 mM) was added. The samples were vigorously mixed for 10 s in a vortex, and the absorbance was measured at 515 nm after 20 min. The results were expressed in Trolox equivalent antioxidant capacity using a Trolox calibration curve (µmol TEAC/g of oil).

## Statistical Analysis

The date were subjected to Multiple Analysis of Variance (MANOVA) by applying Statistica 12 software (StatSoft, Inc., Tulsa, OK). Significant differences among means were determined through Tukey’s Multiple Range Tests. Differences were considered significant at *p* < 0.05.

## Results and Discussion

### Effect of Plant Seed Oils on the Growth of *Y. lipolytica* W29

**Fig. 1:**
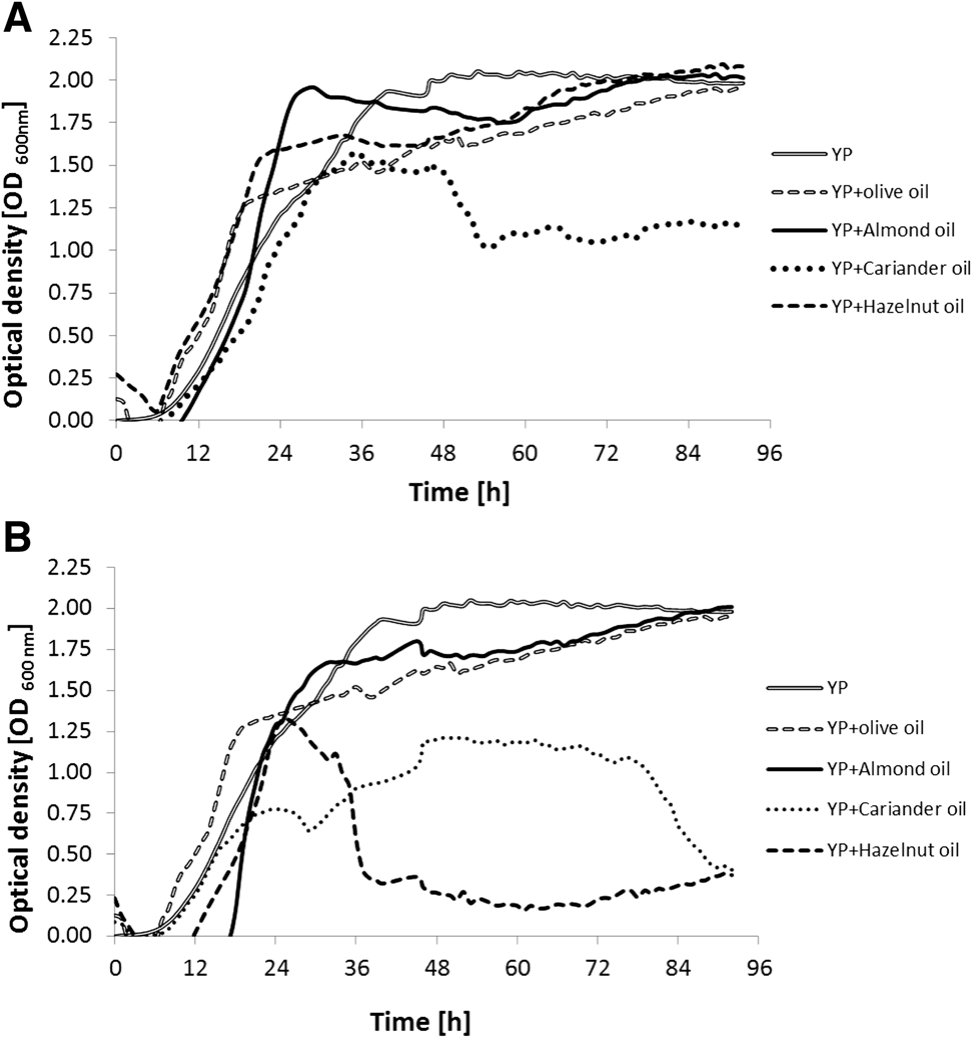
The growth curves of *Y. lipolytica* W29 yeast after the addition to a medium of oils extracted from seeds using Soxhlet method (**a**) and Folch method (**b**)

In order to determine the effect of plant seed oils on the growth of *Yarrowia* yeast, the growth curves of these microorganisms were plotted (96 h cultivation in bioscreen). The results are shown in Fig. 1a, b, but Fig. 1a presents the growth curves of yeast in the media supplemented with plant oils extracted from seeds using the Soxhlet method, and Fig. 1b was obtained using the Folch method. In addition to the curves plotted for tested oils, the figures include yeast growth curves on the substrate supplemented with commercial olive oil and a medium not containing an inducer (YP medium). The addition of both plant seed oils extracted with Soxhlet and Folch methods to the medium enabled the growth of *Y. lipolytica* yeast. In the case of adding the oils extracted with the Soxhlet method, a clear difference may be observed especially for almond and coriander oils (Fig. 1a). The addition of almond oil as a source of carbon into the medium contributed to the most intense growth of *Y. lipolytica*. After 24 h of culture, the value of optical density reached approximately 2.0, while in the medium with the addition of hazelnut and olive oils, this value was reached after 70 and 84 h, respectively. In the medium with coriander oil addition, the propagation of *Yarrowia* cells was much slower. The optical density was 25% lower in comparison with the medium enriched with almond oil. In addition, after 2 days of culture, the cells began to die. After 48 h, the value of optical density decreased from 1.5 to approximately 1.0 (33%).

For substrates containing oils extracted from seeds with the Folch method (Fig. 1b), *Yarrowia* yeast growth curves were arranged a bit differently. In this case, the cells multiplied more slowly. After 24 h of culture, OD values were lower by 0.25 for the medium with almond oil and coriander oil. Moreover, for a medium supplemented with hazelnut oil, dieback of the yeast cells was observed after 24 h. The slower propagation of the yeast on the media supplemented with oils obtained with the Folch method may be due to the procedure for the oil extraction from plant material. A mixture of two solvents: chloroform and methanol, was used in the extraction method acc. to Folch. It is hypothesized that after extraction, these solvents were not completely evaporated and at low concentration might be present in the culture medium. The amount of residual solvents in oils obtained by Folch procedure was, additionally, determined by measuring the mass loss of samples under storage in an oven under vacuum at room temperature for 24 h. The loss of weight was observed at the level of 0.1%. Methanol used in Folch extraction and its oxidation product—formaldehyde, are also toxic to yeast growth [18]. Formaldehyde has been proven to be more toxic than methanol, but the alcohol is known to regulate the production rate of yeast biomass. The mechanism of toxicity against microorganisms has not been tested yet for most organic solvents, but, nevertheless, it is considered that highly toxic organic solvents destroy the integrity of the cell membrane and accumulate in the lipid layer of plasma. Each organism has its own intrinsic tolerance level for organic solvents, which is determined genetically and is also influenced by environmental factors. Log P (the logarithm of the octanol–water partition coefficient) is a parameter that has been used as an indicator of solvent partitioning from the aqueous medium into the membrane of an organism. Lipophilic solvents (log *P* > 4) accumulate preferentially in membranes, but they will not reach a high membrane concentration owing to their low water solubility, and as such are not toxic to an organism. Solvents with log *P* values below 4 are more water soluble and partition well in the membrane. As a result, the actual membrane concentration of these solvents will be relatively high, and these solvents may be toxic to cells. For solvents used in this study, log *P* values are −2.0 and 3.5 for chloroform and *n*-hexane, respectively [19].

**Fig. 2:**
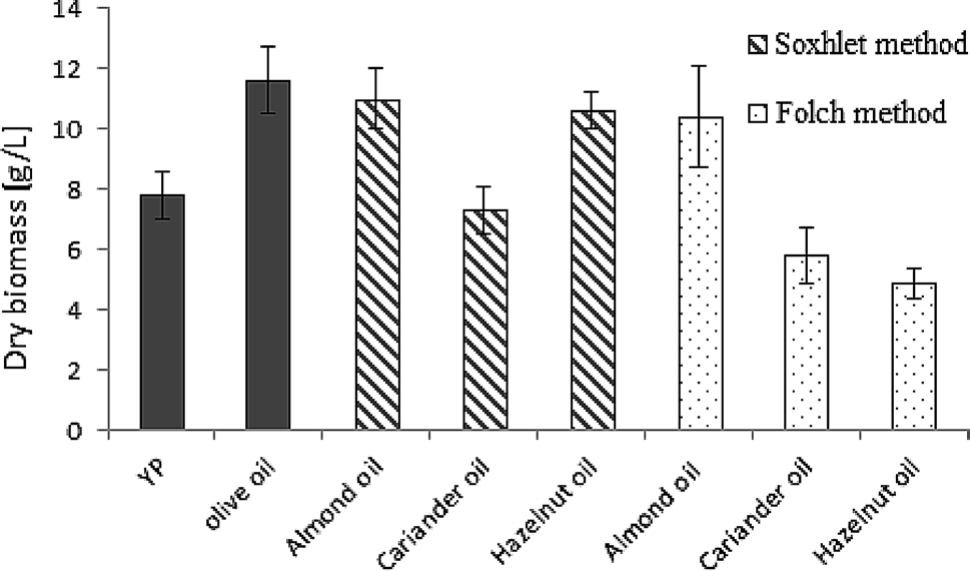
A representative graph for dry biomass production by *Y. lipolytica* W29 on medium enriched with plant seed oils, after 96 h. Each experiment was repeated at least three times (data represent mean ± SD, *n* = *3*)

The presence of residual solvents in oils samples extracted according to the Folch procedure might also be affecting the yield of yeast cell biomass estimated after 96 h of culture in the media when plant seed oils were used as the source of carbon. The yield of dry biomass of *Yarrowia* cells was lower in the media containing plant oils obtained after extraction of seeds by using a mixture of solvents (chloroform/methanol) (Fig. 2). The lowest biomass yield was achieved in the medium with hazelnut oil. In the medium enriched with seed oils extracted with the Soxhlet method, the biomass yield was higher than in the medium without oil but lower than in the medium with olive oil. Using a polar solvent in the oil extraction from seeds, it is also possible to extract compounds with antioxidant and antimicrobial activities [20]. It could be the next factor that affected the growth inhibition of *Y. lipolytica* yeast and the low yield of yeast biomass when the medium was enriched with hazelnut oil extracted with the Folch method.

### Effect of Plant Seed Oils on Lipolytic Activity of *Y. lipolytica* W29

To analyze the effect of plant oils added to the culture medium on *Y. lipolytica*, the lipolytic activity of these microorganisms was determined with the spectrophotometric method. Results shown in Table 1 demonstrate that the plant oils used as a carbon source affected the extracellular lipolytic activity of *Y. lipolytica*. Among plant seed oils tested, the best inducer of lipase turned out to be almond oil, regardless of the extraction method. The presence of this oil in the culture medium resulted in enhanced extracellular lipolytic activity of yeast, achieving maximum values of 2.33 and 2.06 U/mL after 48 h, when the oil used was extracted with the Soxhlet or Folch method, respectively. The lipolytic activity of *Y. lipolytica* in this medium was, however, lower than in the medium containing olive oil. According to the results presented by Niaz *et al.* [21], almond oil was also found to be the best carbon source that improved extracellular lipase production by *Trichophyton* spp. and ensured the maximum lipase activity (73.34 U/mL) as compared to other oils.

**Table 1:** Lipolytic activity of *Y. lipolytica* W29 during growth on medium enriched with plant oils

Extraction method	Sample of plant seed oils	Lipolytic activity of *Y. lipolytica* W29 at different times of growth (U/mL)		
		24 h	48 h	72 h
Soxhlet method (*n*-hexane)	Almond	2.05 ± 0.23aA	2.33 ± 0.19aA	1.78 ± 0.11bA
	Hazelnut	1.65 ± 0.13aA	1.92 ± 0.37aB	1.22 ± 0.42bB
	Coriander	1,40 ± 0.47aB	1.03 ± 0.24bC	1.05 ± 0.40bB
Folch method (chloroform/methanol)	Almond	0.98 ± 0.10aB	2.06 ± 0.21bA	1.86 ± 0.22bA
	Hazelnut	0.65 ± 0.01aC	0.57 ± 0.06bD	0.52 ± 0.11bC
	Coriander	0.40 ± 0.11aC	0.90 ± 0.17bC	0.78 ± 0.15bC
	Olive oil	2.30 ± 0.13aA	3.05 ± 0.18bE	2.17 ± 0.25aD
	Control sample (YP—medium without oils)	0.72 ± 0.04aB	0.81 ± 0.08bC	0.65 ± 0.15cC

Values mean ± standard deviation. Means in the same column with different uppercase letters and in the same row with different lowercase letters are significantly different (*p* < 0.05)

The least effective inducer of lipase proved to be coriander oil. The low biomass yield resulted in a low extracellular lipolytic activity of *Yarrowia*. Its presence in the culture medium affected the lipolytic activity of *Y. lipolytica* that reached 1.03 and 0.90 U/mL after 48 h, respectively, and was approximately twofold lower than the values assayed for the culture medium with the addition of almond oil. The lipolytic activity in the medium with coriander oil after 48 h was comparable to that found in the medium without a lipid carbon source (YP). It is very likely that in the case of coriander oil, depending on the extraction method applied, an important role may be ascribed to the presence of chemical compounds with antimicrobial activity, especially these which are characteristic for essential oils. Essential oils content in coriander seeds is very different and depends on many factors such as origin of cultivars, climatic conditions, geographic position of the growth region, and increases as the fruit ripens [22]. The predominant substance in coriander seed essential oil extracted with the Soxhlet method is linalool. It was reported to have an antibacterial effect against many bacterial strains, and it could be responsible for the antibacterial activity of oils [22]. Generally, the linalool extraction yield increases with temperature increase, and in the case of Soxhlet extraction it is slightly higher compared to the hydrodistillation method, but lower compared to the subcritical water extraction [23]. Other important compounds with the antimicrobial activity present in coriander seed essential oil are γ-terpinene, geranyl acetate, α-thujene, β-pinene, and camphor. It was also found that coriander oil exhibited a significant antibacterial activity against all tested strains of *Acinetobacter baumannii* in different growth phases [24]. The rapid lethality indicated that the bactericidal activity of the oil can be associated with the disruption of cellular walls of the studied strains. It was also possible to observe that cells in the exponential growth phase were more susceptible to coriander essential oil than these in the stationary phase. Silva *et al.* [25] also drew attention to the inhibitory effect of coriander oil in relation to yeast of the genus *Candida*, *Candida albicans,* and *Candida tropicalis*. In turn, the coriander seed oil extracted with the Folch method using a polar solvent such as methanol in the mixture of solvents facilitates the extraction of non-volatile components such as non-volatile polyphenolic compounds and others bioactive components such as sterols and phospholipids. The chemical structure of phenolic compounds, in particular the number and position of substitution in the benzene ring, and the saturated chain length may determine the antimicrobial activity. The antimicrobial effect increases with increasing length of the alkyl chain. Butyl ester is approximately three times more toxic than the methyl ester [26]. Caffeic acid alkyl esters were found to be effective antimicrobial agents against *Escherichia coli* and S*taphylococcus aureus* [27]. Their activity was directly dependent on their lipophilicity, which affected bacterial susceptibility, physicochemical properties of the bacteria, and the integrity of membranes.

### Determination of Oil Extraction Yield and Fatty Acid Composition in Plant Oils

The extraction yield of oils from seeds with the Folch method was found to be most effective compared to the Soxhlet extraction. The oils extracted at room temperature using a mixture of solvents, such as chloroform and methanol, may contain both nonpolar and polar lipids, which may result in the improved yield of their extraction from plant material. In turn, *n*-hexane is an ideal solvent for nonpolar lipids extraction and for the isolation of all boiling range volatile compounds. In our case, the choice of solvent can be significant for oil extraction yield. It was also observed that the oil extraction yield using a Soxhlet extractor at 70 °C was lower in comparison to the oil extraction at room temperature. Although it is well known that the solvent recycle presented by the Soxhlet technique may contribute to a higher solute solubilization, it thus maximizes the extraction yield. The results obtained for almond oil after Soxhlet extraction were lower than those reported by Miraliakbari and Shahidi [28]. Also, chloroform/methanol extracted hazelnut and almond oils were characterized by a lower percentage value of oil extraction yield. In our study, coriander oils were extracted with the yield of 16.60 and 20.32% using *n*-hexane and a mixture of solvents, respectively. Msaada *et al.* [29] presented changes in coriander oil yield during maturation of fruits cultivated in two regions of Tunisia. In both regions, at full fruit ripeness, the oil content was maximal while differences were observed between 5 and 13 days after flowering for Oued Beja fruits reaching values from 2.70 to 22.60%. Among oil samples analyzed in our study, the highest extraction yield was observed for hazelnut oil and the lowest one for coriander oil, regardless of the extraction method applied. Generally, nuts have a high total fat content, ranging from 46% in pistachios to 76% in macadamia nuts [30]. According to the Ros [30] studies, the content of fat in hazelnuts and almonds was 60.8 and 50.6 g per 100 g, respectively. The coriander seeds are also a rich source, however, they are poorer than hazelnuts and almonds.

To identify the causes of different lipolytic activity of *Y. lipolytica* W29 yeast propagated on the culture media with the above-mentioned oils, the oils were determined for fatty acid composition (Table 2). Generally, the oils extracted using *n*-hexane (Soxhlet method) had fatty acid composition comparable to the oils obtained by seed extraction with a mixture of solvents—chloroform/methanol (Folch method). Thirteen fatty acids were identified, where stearic acid (C18:0), oleic acid (C18:1, *n*-*9*), and linoleic acid (C18:2, *n*-*9*) were the major fatty acids regardless of the extraction method. Their percentage composition ranged from 2.65 to 6.09 for stearic acid, from 3.48 to 79.42 for oleic acid, and from 12.01 to 24.82 for linoleic acid. In plant seeds oils, saturated fatty acids constituted from 2.81 to 6.20% of the total fatty acids, monounsaturated fatty acids were in the range from 65.39 to 81.24%, and polyunsaturated fatty acids accounted from 11.95 to 25.33% of total fatty acids. The highest content of saturated fatty acids (C18:0 and C20:0) was found in hazelnut oil. Among them, palmitic acid was identified but only in coriander oil extracted with the Folch method. The contents of oleic acid assayed in hazelnut oil were higher than those found in almond and coriander oils. However, linoleic acid—as a representative of polyunsaturated fatty acids—was present in a significantly higher amount in almond oil than in hazelnut and coriander oils. In addition, high concentrations of petroselinic acid (C18:1, *n*-12), accounting for 71.31 and 72.55% of total fatty acids, were found in coriander oil obtained with the Soxhlet and Folch methods, respectively. Petroselinic acid enables producing chemical derivatives different from those that can be produced from other oils [31].

**Table 2:** Fatty acids composition of plant seed oils obtained by Soxhlet and Folch methods

Fatty acids (%)	Sample of plant oils						
	Soxhlet method (*n*-hexane)			Folch method (chloroform/methanol)			
	Almond	Coriander	Hazelnut	Almond	Coriander	Hazelnut	Olive oil
C16:0	–	–	–	–	2.01 ± 0.04	–	9.31 ± 0.06
C18:0	5.58 ± 0.02	2.81 ± 0.02	6.00 ± 0.04	5.98 ± 0.03	2.65 ± 0.03	6.09 ± 0.02	3.84 ± 0.03
C20:0	–	–	0.11 ± 0.01	0.06 ± 0.01	–	0.11 ± 0.01	0.39 ± 0.01
SFA	5.58 ± 0.02	2.81 ± 0.02	6.11 ± 0.06	6.04 ± 0.05	4.66 ± 0.07	6.20 ± 0.04	13.54 ± 0.05
C16:1n-7	0.43 ± 0.03	0.47 ± 0.04	0.26 ± 0.05	0.47 ± 0.03	0.50 ± 0.02	0.26 ± 0.03	0.72 ± 0.02
C17:1	0.10 ± 0.02	–	–	0.13 ± 0.02	–	–	0.13 ± 0.01
C18:1n-12	–	71.31 ± 0.06	–	–	72.55 ± 0.05	–	–
C18:1n-9	63.72 ± 0.05	3.48 ± 0.02	78.09 ± 0.04	67.80 ± 0.06	5.47 ± 0.04	79.42 ± 0.04	76.32 ± 0.05
C18:1n-7	1.14 ± 0.02	0.76 ± 0.04	1.42 ± 0.03	–	0.74 ± 0.04	1.40 ± 0.05	–
C20:1n-9	–	–	0.15 ± 0.03	–	–	0.16 ± 0.04	0.43 ± 0.02
MUFA	65.39 ± 0.09	76.02 ± 0.06	79.92 ± 0.07	68.40 ± 0.08	79.26 ± 0.07	81.24 ± 0.06	77.60 ± 0.07
C18:2n-6	23.03 ± 0.06	13.92 ± 0.05	11.85 ± 0.04	24.82 ± 0.07	14.35 ± 0.04	12.01 ± 0.05	8.21 ± 0.04
C18:3n-3	–	0.16 ± 0.02	0.10 ± 0.01	–	0.17 ± 0.03	0.10 ± 0.01	0.60 ± 0.02
C22:6n-3 (DHA)	1.71 ± 0.06	–	–	–	–	–	–
C18:2n-*6* (CLA2^a^)	0.59 ± 0.06	–	–	–	–	–	–
PUFA	25.33 ± 0.08	14.08 ± 0.05	11.95 ± 0.05	24.82 ± 0.07	14.52 ± 0.06	12.11 ± 0.07	8.81 ± 0.04

Results are given as the average of triplicate determination ± standard deviation. *SFA* are total saturated fatty acids, *MUFA* are total monounsaturated fatty acids, *PUFA* are total polyunsaturated fatty acids

– not identified

^a^ *CLA2* = conjugated linoleic acid (*trans*-10,*cis*-12-C18:2n-*6*)

Compared with literature data, the content of unsaturated fatty acids in hazelnut oil was slightly lower than the values found by Kirbaşlar and Erkman [32], and was very similar to data reported by Savage *et al.* [33], especially when oil was extracted from hazelnut cultivars such as Whiteheart, Tonda di Giffoni and Campanica. However, linoleic acid content in hazelnut oil was found to be higher compared to literature data [34]. In the case of almond oil, results of our study on oleic acid levels are in agreement with those reported by Moayedi *et al.* [35]. In commercial almond cultivars grown in various regions, oleic and linoleic acids accounted for about 90% of the total lipids. However, Kirbaşlar *et al.* [36] demonstrated slight differences in the fatty acid profile in almond oil extracted with *n*-hexane. The content of oleic acid was higher than in the present study, while the levels of stearic and linoleic acids were lower. Almond oil contained also docosahexaenoic acid (DHA, C22:6, *n*-*3*) which is mainly present in oils: cod liver, herring, salmon, and algae. Beyhan *et al.* [37]. also reported the presence of DHA in almond but in 17-4 species at 0.81% and in 300-1 species at 0.04%.

### Effect of Fatty Acid Composition on Lipolytic Activity of *Y. lipolytica* W29

Considering the fatty acid composition in oils used in the present study, it was found that unsaturated fatty acids, mainly oleic and linoleic acids, might in some way affect the lipolytic activity of *Y. lipolytica* W29. Among all tested substrates, the highest lipolytic activity was shown for the supernatant from the commercial olive oil medium (Table 1). Its lipolytic activity was by 11 (after 24 h), 24 (after 48 h) and 32% (after 72 h) higher than in the medium containing oil extracted from almonds with the Soxhlet method and by 57 (after 24 h), 32 (after 48 h) and 14% (after 72 h) higher compared to the medium with almond oil obtained with the Folch method. Olive oil contained 86.41% of unsaturated fatty acids, including 76.32% of oleic acid and 8.21% of linoleic acid, whereas the content of oleic acid in almond oil was lower, and that of linoleic acid was the highest among all the tested oils. Oleic acid present in olive oil also played a crucial role in extracellular lipases production by *Y. lipolytica* DSM 3286 and acted as a stabilizer/activator of these enzymes in the Darvishi *et al.* studies [9]. The likely reason is that an expression system containing the *LIP2* gene expressed and the inducible *POX2* promoter were under the control of oleic acid. Among various lipids and fatty acids analyzed by Thabet *et al.* [38], olive oil was also found to be the best inducer of lipase production by *Sporobolomyces salmonicolor,* but a lower activity was observed for pure oleic acid though its impact on the biomass yield was good. Kabara *et al.* [39] indicated that monoenoic acid (C18:1) was more inhibitory against the studied organisms than saturated fatty acids, but was less active than dienoic derivatives (C18:2). Speert *et al.* [40] showed that oleic acid was not effective against *S. aureus* and *E. coli* strains but more active against group A *Streptococci*. Among a series of free fatty acids studied, only oleic acid inhibited the growth of a number of Gram-positive bacteria including hospital and community-associated MRSA [41]. In our study, the effect of almond oil as an inducer of lipase synthesis was lower than of the olive oil. It may also be caused by the presence of antimicrobial and toxic substances in this oil such as benzaldehyde or hydrocyanic acid (prussic acid) [9].

In our study on the lipolytic activity of *Y. lipolytica*, coriander oil turned out to be the least effective inducer. Although it was a source of unsaturated fatty acids, its main representative was not oleic acid but petroselinic acid differing in the position of the double bond in the carbon chain. On the other hand, despite being similar to olive oil composition of unsaturated fatty acids, the hazelnut oil showed a lower extracellular lipolytic activity of *Y. lipolytica* W29. In this case, a high content of oleic acid did not correspond with lipase activity. There are also other factors that are likely to affect the activity of extracellular lipolytic enzymes. They have not as yet been precisely identified, but Fabiszewska and Białecka-Florjańczyk [42] presented several hypotheses. The activation of lipase expression may be related to the accumulation of specific fatty acids inside yeast cells, whereas *de novo* synthesis of lipid compounds is insufficient. The presence of Ca^2+^, Mg^2+^, Zn^2+^, Cu^2+^ or Ni^2+^ ions in oils may also be an important factor, which can determine whether an individual vegetable oil stimulates microbial lipase production.

### Total Phenolic Content and DPPH Radical Scavenging Activity

The total phenolic content of oils obtained from almonds, hazelnuts and coriander seeds is shown in Table 3. The highest contents of total phenolic compounds were found in coriander oils extracted from seeds using both methods—Folch and Soxhlet (210.12 and 165.08 µg GA/g of oil, respectively). Hazelnut and almond oils showed no significant differences in the content of total polyphenols. Their content in almond oil extracted with chloroform/methanol was slightly higher than in that extracted with *n*-hexane. However, hazelnut oil extracted with *n*-hexane was characterized by a slightly higher content of total phenolics than the oil extracted with chloroform/methanol solvents.

**Table 3:** Total phenolic content and antioxidant activity in plant oils measured by the DPPH method

Sample of plant seed oils	Total phenolic content (µg GA/g oil)	DPPH methanolic extract (µmol TEAC/g oil).	DPPH plant oil (µmol TEAC/g oil)
Soxhlet method (*n*-hexane)			
Almond	14.23 ± 0.98a	0.21 ± 0.03a	0.42 ± 0.04a
Coriander	165.08 ± 7.07b	1.16 ± 0.13e	2.15 ± 0.18c
Hazelnut	19.12 ± 1.33a	0.53 ± 0.09d	3.00 ± 0.25d
Folch method (chloroform/methanol)			
Almond	19.34 ± 5.07a	0.36 ± 0.06b	0.46 ± 0.07a
Coriander	210.12 ± 8.40c	3.51 ± 0.37f	5.16 ± 0.24e
Hazelnut	15.46 ± 0.82a	0.45 ± 0.08c	1.38 ± 0.16b
Olive oil	235.11 ± 0.72c	1.55 ± 0.21e	2.67 ± 0.23cd

Results are given as the average of triplicate determination ± standard deviation. Means with different letters in the same column differ significantly, *p* < 0.05

Table 3 shows also DPPH values of the methanolic extracts of the studied oils and antioxidant activity measured with the DPPH assay in seed oil samples. The DPPH radical scavenging activity of all samples ranged between 0.21 and 5.16 µmol TEAC/g of oil, and its highest value was assayed in the methanolic extracts from coriander seed oils. The methanolic extract from almond oil exhibited the lowest ability to scavenge DPPH radicals. The order of the effectiveness of oils extracted from seeds with chloroform/methanol in quenching DPPH radicals was as follows: coriander oil > hazelnut oil > almond oil. Coriander oil extracted with the Folch method was a more potent quencher compared to that extracted with *n*-hexane and olive oil. However, hazelnut oil extracted from nuts with *n*-hexane showed a higher radical scavenging activity than that extracted with chloroform/methanol. These results indicate that the chloroform/methanol extraction enabled obtaining oils with a higher content of total phenols and a greater DPPH radical scavenging capacity. Similar antioxidant activity trends were also observed by Aranz *et al.* [34].

### Effect of Total Phenolic Content on Lipolytic Activity of *Y. lipolytica* W29

The content of polyphenols in plant seed oils and their antioxidant activity may also affect the lipolytic activity of yeast multiplied on different lipid carbon sources. Our data showed that when the content of phenolic compounds in plant oils was higher, the lipolytic activity and yeast biomass yield decreased. Therefore, the lowest lipolytic activity was found in the medium with the addition of coriander oil, which was also characterized by the high radical scavenging activity towards DPPH radical. Phenolic compounds are widely distributed in plants and may also exhibit bactericidal and fungicidal activities. The 2,4-Dihydroxybenzoic and protocatechuic acids were the phenolic compounds with a higher activity against the majority of Gram-negative and Gram-positive bacteria. In the Lou *et al.* [43] studies, chlorogenic acid was active against *treptococcus pneumoniae* by provoking irreversible permeability changes in the cell membrane, causing cells to lose the ability to maintain the membrane potential and cytoplasm macromolecules including nucleotides. A higher content of phenolic compounds in hazelnut oil and its better ability to scavenge DPPH radicals compared to almond oil were probably the cause of a slower proliferation of *Yarrowia* yeast cells and a lower lipase activity, despite the fact that it was characterized by a higher content of oleic acid. Generally, oils extracted with the Folch method demonstrated a relatively lower average lipolytic activity than their *n*-hexane counterparts because of a higher content of phenolic compounds and antioxidant activity of these oils, except for hazelnut oil. The inhibitory effect of phenolic compounds on lipase (porcine pancreatic lipase) was mentioned by Hadrich *et al.* [44].

## Conclusions

Our results concerning the application of plant oils as a carbon source to determine the growth of *Y. lipolytica* W29 and its lipolytic activity are promising and show that plant oils might be renewable low-cost substrates used for lipase synthesis. Of key significance was the method of oil extraction. The more beneficial effect on the growth of *Y. lipolytica* and the lipolytic activity of this microorganism was achieved when the culture medium contained the oils extracted from plant seeds with *n*-hexane as a solvent (Soxhlet method) in comparison to their counterparts extracted using mixture of solvents such as chloroform/methanol (Folch method). The best inducer of lipases among the tested oils, obtained by using *n*-hexane, turned out to be almond oil. Its effect on the synthesis of *Y. lipolytica* extracellular lipases was lower after 48 h of culture and close to the value received after 24 h of culture compared to olive oil that is often used as a hydrophobic carbon source. Almond oil was also a rich source of unsaturated fatty acids, especially linoleic acid. The presence of fatty acids with a double bond in the carbon chain in the oils might be an important factor which determined the lipolytic activity of *Y. lipolytica* W29. Plant oils extracted with *n*-hexane were also characterized by a lower content of phenolic compounds and weaker antioxidant activity when compared to their counterparts extracted using the Folch method. On the other hand, the extraction yield of oils obtained from almond and hazelnut seeds with a polar solvents was slightly higher than the oils extracted with *n*-hexane.
